# Microglia Activation as a Biomarker for Traumatic Brain Injury

**DOI:** 10.3389/fneur.2013.00030

**Published:** 2013-03-26

**Authors:** Diana G. Hernandez-Ontiveros, Naoki Tajiri, Sandra Acosta, Brian Giunta, Jun Tan, Cesar V. Borlongan

**Affiliations:** ^1^Department of Neurosurgery and Brain Repair, Center of Excellence for Aging and Brain Repair, Morsani College of Medicine, University of South FloridaTampa, FL, USA; ^2^James A. Haley Veterans Administration HospitalTampa, FL, USA; ^3^Rashid Laboratory for Developmental Neurobiology, Silver Child Development Center, Department of Psychiatry and Behavioral Neurosciences, Morsani College of Medicine, University of South FloridaTampa, FL, USA

**Keywords:** head trauma, microglia, inflammatory response, secondary cell death, anti-inflammatory therapy, brain imaging

## Abstract

Traumatic brain injury (TBI) has become the signature wound of wars in Afghanistan and Iraq. Injury may result from a mechanical force, a rapid acceleration-deceleration movement, or a blast wave. A cascade of secondary cell death events ensues after the initial injury. In particular, multiple inflammatory responses accompany TBI. A series of inflammatory cytokines and chemokines spreads to normal brain areas juxtaposed to the core impacted tissue. Among the repertoire of immune cells involved, microglia is a key player in propagating inflammation to tissues neighboring the core site of injury. Neuroprotective drug trials in TBI have failed, likely due to their sole focus on abrogating neuronal cell death and ignoring the microglia response despite these inflammatory cells’ detrimental effects on the brain. Another relevant point to consider is the veracity of results of animal experiments due to deficiencies in experimental design, such as incomplete or inadequate method description, data misinterpretation, and reporting may introduce bias and give false-positive results. Thus, scientific publications should follow strict guidelines that include randomization, blinding, sample-size estimation, and accurate handling of all data (Landis et al., 2012). A prolonged state of inflammation after brain injury may linger for years and predispose patients to develop other neurological disorders, such as Alzheimer’s disease. TBI patients display progressive and long-lasting impairments in their physical, cognitive, behavioral, and social performance. Here, we discuss inflammatory mechanisms that accompany TBI in an effort to increase our understanding of the dynamic pathological condition as the disease evolves over time and begin to translate these findings for defining new and existing inflammation-based biomarkers and treatments for TBI.

## Introduction

**Figure 1:**
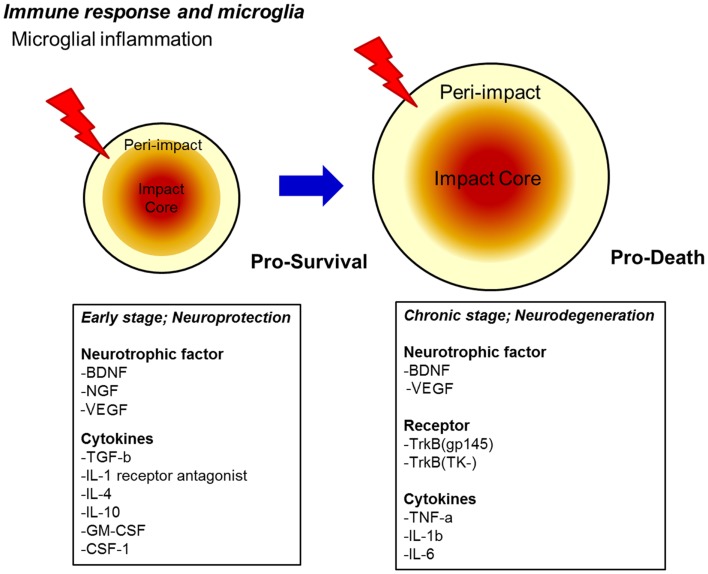
**Proposed secondary cell death mechanism after TBI**. A sensitive balance in neurotrophic factor secretion and cytokine expression dictates the fate of injured neurons towards pro-survival (early stage) and pro-death (chronic stage) cellular processes after TBI. Upregulation of neurotrophic factors and cytokines may renderneuroprotectionin the early stage, whereas downregulation of neurotrophic factors coupled with continued upregulation of specific pro-death cytokines, as well as the onset of oxidative stress, massive edema, and alterations in endogenous neurotransmitter function may promote neurodegeneration in the chronic stage.

Traumatic brain injury (TBI) is characterized by a damage to the brain as a result of a violent impact, blow or jolt to the head that causes the brain to strike the inside of the skull or when an object perforates the skull and reaches brain tissue. The most recent estimates of the incidence and prevalence of TBI indicate that each year 235,000 Americans are hospitalized for non-fatal TBI, 1.1 million are treated in emergency, and 50,000 die (Corrigan et al., 2010). Common features of TBI include bruising, torn tissues, bleeding, and physical damage to the brain resulting in long term complications or death. It can be classified based on its severity, anatomical areas affected, and causative forces. Depending on the extent of damage to the brain, TBI varies from mild to moderate to severe. Serious secondary events may also occur, such as oxidative stress, massive edema, and alterations of endogenous neurotransmitter mechanisms, as depicted in Figure 1. In the case of mild TBI, the patient may remain conscious or faint for a few seconds or minutes. Characteristic symptoms of mild TBI include headache, confusion, lightheadedness, dizziness, blurred vision or tired eyes, ringing in the ears, bad taste in the mouth, fatigue or lethargy, a change in sleep patterns, behavioral or mood changes, and trouble with memory, concentration, attention, or thinking (National Institute of Neurological Disorders and Stroke, National Institutes of Health). In moderate to severe TBI similar symptoms may occur, but with worse manifestations. For example headaches may become intermittent, repeated vomiting or nausea, seizures, inability to awaken from sleep, dilation of one or both pupils of the eyes, slurred speech, weakness or numbness in the extremities, loss of coordination, and increased confusion, restlessness, or agitation. War-related TBI is usually associated with injury to the brain due to an improvised explosive device (IED) blast during military conflicts (Bogdanova and Verfaellie, 2012; Duckworth et al., 2012; Goeller et al., 2012). When a frontal blast wave encounters the head, a shock wave is transmitted through the skull, cerebrospinal fluid (CSF), and tissue, causing negative pressure at the contrecoup that may result in cavitation (Goeller et al., 2012).

Patients with varying severity of TBI often struggle with physical and cognitive impairments for months, or years; and some never reach full recovery. An estimated 43.3% of Americans have residual disability 1 year after injury (Corrigan et al., 2010). Although TBI is typically believed to be a static pathological insult from a single event, previously unrecognized clinical symptoms can arise many years after the initial injury (Giunta et al., 2012). The most recent estimate of the prevalence of US civilian residents living with disability following hospitalization with TBI is 3.2–5.3 million (Corrigan et al., 2010; Coronado et al., 2011). Thus, greater efforts should center on this sector of the population living and aging with post-TBI sequelae.

In this mini-review, we bring attention to microglia, which possess a double-edge sword function, in that microglia can mount both pro-survival and pro-death actions after TBI occurrence. We propose new and present inflammation-based biomarkers that may enhance regenerative abilities and decrease degenerative events associated with microglial response for the treatment of TBI. Microglia exert neuroprotection by sequestering, via phagocytosis, foreign bodies that aberrantly penetrate the brain (Noda et al., 2011; Voss et al., 2012). Unfortunately during aging, microglial cells also display reduced phagocytic capacity (Fiala et al., 2005; Kohman, 2012). The challenge for developing therapies targeting microglial function is to manipulate microglia activation toward a reparative process that could retard, or even halt the progressive pathological symptoms of TBI and its co-morbidity factors.

## Immune Response and Microglia

Activation of the immune system in the central nervous system (CNS) has become increasingly recognized as a key component of the normal process of aging, but also of the pathological onset and progression of many neurological disorders including TBI and neurodegenerative diseases. There are three phenotypic states of microglia-based on developmental and pathophysiologic studies: (*i*) resting, ramified; (*ii*) activated non-phagocytic [or antigen presenting cell (APC)-like] found in areas involved in CNS inflammation; and (*iii*) reactive, in which phagocytic microglia is present in areas of trauma or infection (Frei et al., 1987; Suzumura et al., 1987; Williams et al., 1992; Panek and Benveniste, 1995; Walker et al., 1995). With respect to activation, macrophages and microglia are able to polarize into two major subtypes, categorized as M1 or M2 (Mosser, 2003; Gordon and Taylor, 2005). This “classical” or *M1* subtype excessively secretes proinflammatory cytokines and promotes cell-mediated immunity (Mosser, 2003; Gordon and Taylor, 2005). It is marked by production of high levels of interferon-gamma (IFN-γ), tumor necrosis factor (TNF)-α, interleukin (IL)-1, IL-12, and low levels of IL-10. The *M1* phenotype may be activated when microglia contact HIV proteins (such as transactivator of transcription [Tat] bind toll-like receptors 3 or 4 as well) (Suh et al., 2009). “Alternatively activated” or *M2* microglia tend to dampen (Bruce-Keller et al., 2001) inflammation, clear cellular debris (including amyloid plaques), and produce very low levels of TNF-α, IL-1, IL-12, and high amounts of anti-inflammatory IL-10 and transforming growth factor (TGF)-β, and suppressor of cytokine signaling (SOCS) (Mosser, 2003; Gordon and Taylor, 2005; Qin et al., 2006; Akhtar et al., 2010). These two phenotypes, respectively, belong to the type *ii* or *iii* microglial states. Further, the factors which cause polarization to *M1* or *M2* reinforce the maintenance of that phenotype in a cycle-like manner.

The initial inflammatory response after TBI results in neuronal injury and disruption of the blood-brain barrier (Smith et al., 1997; Nagamoto-Combs et al., 2007; Namas et al., 2009). Microglial cells become activated within minutes, and resemble peripheral macrophages by acting as APCs releasing proinflammatory cytokines and chemokines (Town et al., 2005; Cao et al., 2012). Activated microglia also produce other neurotoxic products after injury such as nitric oxide (NO) and superoxide free radicals that generate reactive oxygen species (ROS) and reactive nitrogen species (RNS). In animal models of cortical controlled impact (CCI); fluid percussion brain injury in rats; combined unilateral lesion of the primary motor cortex and of the lateral pre-motor cortex in rhesus monkeys, microglial cells remain in their activated state for at least 1 year, especially in the thalamic area (Smith et al., 1997; Nagamoto-Combs et al., 2007; Nagamoto-Combs and Combs, 2010; Jacobowitz et al., 2012; Jin et al., 2012). Human postmortem studies have shown microglial activation 17 years after TBI in subcortical brain areas (Ramlackhansingh et al., 2011). These accrued results suggest the persistence of a chronic inflammatory stage mediated by microglia.

**Figure 2:**
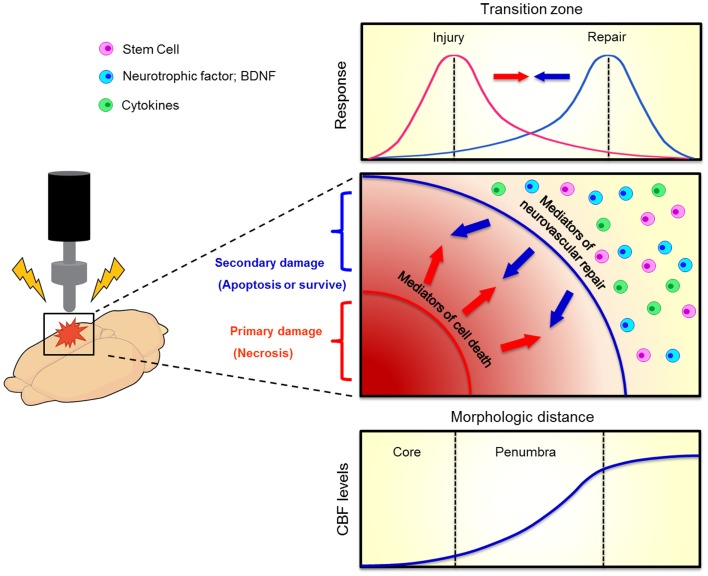
**Evolution of penumbra after TBI**. The brain tissue surrounding the impacted core of TBI can become vulnerable to cell death due to spreading waves of pro-death cytokine mediators. This at-risk brain tissue corresponds to the penumbra which comprises the transition zone between injury and repair (top graphics). A therapeutic window exists for the repair process to abrogate the injury progression. When the brain cell faces damage, it suffers from two kinds of injuries, namely primary (necrosis) and secondary (apoptosis) cell death (middle graph). Neurovascular repair, such as transplantation of stem cells, upregulation of neurotrophic factors, and inhibition of pro-death cytokines, can rescue against the secondary cell death. The penumbra is traditionally defined as an area with mild to moderate reductions in cerebral blood flow (CBF, bottom graph). Such evolution of penumbra after brain injury was originally observed in stroke (Lo, 2008).

A novel feature of activated microglial cells is the delicate cytokine profile they acquire upon brain insult. Microglial cells may share common markers for activated macrophages including CD68, CD45, and major histocompatibility complex II (MHC-II) (Town et al., 2005; Cao et al., 2012). The sensitive balance in cytokine expression may dictate the fate of injured neurons toward pro-survival or pro-death mechanisms, as illustrated in Figure 2.

Microglial cells exist in at least two functionally distinguishable states once activated – namely a phagocytic phenotype (innate activation) or the aforementioned antigen presenting phenotype (adaptive activation) that is seen post-TBI (Town et al., 2005; Giunta et al., 2012). When injury to the CNS occurs, activated microglial cells acquire a predominant proinflammatory profile. If microglia cells are challenged with certain pathogen-associated molecular patterns (particularly CpG-DNA), they activate a mixed response characterized by enhanced phagocytosis and proinflammatory cytokine production, as well as adaptive activation of T cells (Giunta et al., 2012). Among the repertoire of proinflammatory cytokines, IL-1-β and TNF-α play a pivotal role before, during, and after microglia activation. Once secreted, these cytokines can bind specific receptors to increase the amount of inducible nitric oxide synthase (iNOS). Also, they can act as molecular inducers of programmed cell death or apoptosis as shown in animal and human studies of neurodegenerative diseases like Alzheimer’s disease (AD) (Heneka et al., 1999; Venters et al., 2000; Combs et al., 2001). In addition, after severe TBI, it has been shown that there is a pronounced increase of IL-6, IL-8, TNF-α, and IL-1-β mRNA in human post mortem tissue (Frugier et al., 2010).

Microglia not only express a gamut of proinflammatory cytokines (IL-1, IL-6, TNF-α), but also secrete a myriad of anti-inflammatory cytokines (IL-1 receptor antagonist, IL-4, IL-10) and neurotrophic factors brain-derived neurotrophic factor (BDNF, NGF, TGF-β). Of note, these neurotrophic factors are not exclusively secreted by activated microglia cells or macrophages; indeed these neurotrophic factors are synthesized and secreted by a myriad of cells (i.e., stroma cells, T cells, and astrocytes) within the CNS during inflammatory conditions (Murphy et al., 1977; Pál et al., 2012). Cytokines are small proteins expressed/secreted by microglia under inflammatory conditions. Important exogenous factors capable of cytokine induction in microglia are viral envelope proteins, bacterial cell wall components such as, lipopolysaccharides (LPS) and leukotriene A (LTA); also bacterial DNA, and prions (Heppner et al., 2001). Likewise, endogenous inducers act as inflammatory mediators, such as platelet-activating factor (PAF), lipids, serum proteins, or complement factors; additionally, disturbances in ATP and [K^+^] levels may cause microglia activation (Hanisch, 2002). Thus, shifting the cytokine profile of microglia toward a pro-survival phase (anti-inflammatory cytokines) may increase neuroprotection and regeneration of the CNS after TBI. In the subsequent sections, we discuss both pro-survival and pro-death functions of microglia and identify avenues for therapeutic development, as well as to propose potential biomarker approaches that will maximize the dynamic features of microglia. In the end, a multi-pronged strategy focusing on microglial function may reveal novel therapies and biomarkers for a better understanding of TBI treatment and pathology.

## Harnessing Microglial Pro-Survival Functions

Strategies designed to target specific molecules may be able to manipulate the cytokine profile of the activated microglia. There are several molecules that may be suitable to promote neuronal survival after damage. Among the candidates, granulocyte-macrophage colony stimulating factor (GM-CSF), and colony stimulating factor 1 (CSF-1) have demonstrated to be potent stimuli for microglia *in vitro* (Sawada et al., 1990; Suzumura et al., 1990; Streit et al., 2000). With the proper signaling cues, microglia may shift toward producing more of the neuroprotective substances (e.g., IL-10, IL-1ra, and TGF-β) soon after injury. The direct benefit of these molecules is immediate suppression of proinflammatory cytokines. TGF-β also has been shown to exert neuroprotective effects after injury, including improved function, decreased lesion size, and reduced iNOS production (Hamada et al., 1996; Tyor et al., 2002).

We propose that potential activated microglia-related biomarkers should possess the following features: (a) have high specificity and sensitivity for activated microglia, (b) stimulate microglia to polarize into the M2 phenotype, (c) promote synaptic, neuronal plasticity, and cell survival at a close or distal range from the area of injury, and (d) reduce the inflammatory response post-TBI (Mosser, 2003; Gordon and Taylor, 2005; Ben Achour and Pascual, 2010). Potential microglia-related biomarker to which above criteria could be applied to stimulate microglia into the M2 phenotype may be the chemokine fractalkine, CX3CL1, and its receptor CX3CR1. Both are constitutively expressed in the nervous system. The ligand is expressed exclusively by neurons and endothelial cells, whereas the receptor is s expressed by microglia, astrocytes, and neurons (Rancan et al., 2004). *In vitro* and *in vivo* models of neurological conditions and brain inflammation also reported how CX3CL1 reduces microglia toxicity and, consequently, neuronal damage (Zujovic et al., 2000; Mizuno et al., 2003; Cardona et al., 2006; Bhaskar et al., 2010; Noda et al., 2011; Pabon et al., 2011). Interestingly enough, CX3CL1 and CX3CR1 reduce brain damage in a rodent ischemic model via an adenosine-dependent mechanism (Cipriani et al., 2011). Microglial activation should not be linked only to deleterious effects. There are instances where activated microglia may have a protective role in TBI (Urrea et al., 2007). Case in point, at 3 h after moderate fluid percussion in rats, a new population of cells was recognized in the sub-ventricular zone of the traumatized hemisphere (Urrea et al., 2007). Double labeling confocal microscopy showed newly formed astrocytes, oligodendrocytes, and neurons co-localized with macrophages/microglia even after days on injury. These findings suggest that TBI stimulates a widespread cellular proliferation after injury, and that microglial activation may be involved in the observed focal neurogenesis in the dentate gyrus of the hippocampus (Urrea et al., 2007).

Most TBI animal studies indicate that extended microglial activation at the focal site of injury becomes detrimental over time (Hanisch and Kettenmann, 2007; Ramlackhansingh et al., 2011; Cao et al., 2012; Mannix and Whalen, 2012). Nevertheless, equally compelling studies suggest that persistent microglial activation in regions remote from focal injury might promote brain repair (Nagamoto-Combs et al., 2007, 2010; Thiel et al., 2010), possibly via neurotrophic factor secretion, especially BDNF, proximal and distal to the injured tissue (Krueger et al., 2011; Rostami et al., 2011; Cekic et al., 2012; Colak et al., 2012; Ma et al., 2012; Shi et al., 2012). For instance, in non-human primates even 6 months after injury microglial cells continue to release BDNF and its receptor subtypes TrkB[gp145] and TrkB[TK-] around the cortical lesion site and in the spinal cord (Nagamoto-Combs et al., 2007). Double labeling studies showed that a subpopulation of CD68-immunoreactive microglia/macrophage co-expressed BDNF in the cortex and spinal cord, and also TrkB[gp145] or TrkB[TK-] in the spinal cord; whereas cytokine expression of TNF-α, IL-6, and IL-1-β was less prominent at the 1, 6, and 12-month intervals, suggesting that immediate inflammatory responses had subsided (Nagamoto-Combs et al., 2007). Yet, in a CCI rat model there is a decline in BDNF-mRNA and protein levels measured at 1–14 days post injury (Schober et al., 2012). Moreover, specific BDNF polymorphisms may not be involved in TBI pathology (Bagnato et al., 2012). Notwithstanding these inconsistencies, the findings are encouraging because they suggest that the prolonged microglial activation plays an important role in neurotrophic/tropic signaling, and identifying the appropriate growth factors (i.e., BDNF polymorphism) should facilitate in recovery process of the TBI brain. Thus, more studies are warranted to decipher the molecular cues released by activated microglia proximal and distal to the site of injury, and nurture such therapeutic molecules to be robustly and stably expressed not only at these brain regions vulnerable to secondary cell death, but also to the major impacted brain areas in order to sequester the extent of injured brain after TBI.

## Increasing Phagocytic Activity of Microglia

An equally appealing function of microglia is their phagocytic activity which may rescue neurons from degeneration. Enhancing the phagocytic state of microglia at early stage post-TBI may retard cell death signals to spread to damaged neurons and neighboring cells (Jeon et al., 2012; Schafer et al., 2012; Tamashiro et al., 2012). Furthermore, there are many studies that have documented the process of cell autophagy as neuroprotective after TBI (Clark et al., 2008; Lai et al., 2008; Liu et al., 2008; Venkatesan et al., 2010). Indeed, nearby neurons and astrocytes close to the site of injury are capable of clearing out cell debris after brain injury (Zhang et al., 2008; Loov et al., 2012). An increase in autophagosomal formation proteins, such as microtubule-associated protein 1 light chain 3 (LC3) and beclin 1, has been detected in neurons and astrocytes at 1-h, 3-h, 32 days post-TBI (Zhang et al., 2008). However, autophagy may exacerbate the pathological manifestations of TBI (Bigford et al., 2009; Luo et al., 2011), likely due to aberrant clearance of healthy cells in addition to degenerating cells. Accordingly, for the phagocytic activity of microglia to attenuate the progression of TBI pathology, a regulatory mechanism should be devised to enhance the therapeutic autophagy, while blocking its deleterious side effects.

A potent approach to manipulating microglial phagocytic function is by stimulating neural progenitor cells (NPCs). Mouse NPCs possess a secretory protein profile distinct from other brain cells; specifically proteins that modulate microglial activation and phagocytosis (Mosher et al., 2012). That a close modulatory interaction exists between these two cell types at the protein level suggests that microglia and NPCs may influence each other functions and activity. Some of the factors secreted in large amounts by NPCs include tissue inhibitor of metalloproteinase type-1 (TIMP-1), vascular endothelial growth factor (VEGF), and haptoglobin, which are well known immunomodulatory proteins or regulators of microglia (Forstreuter et al., 2002; Hanisch and Kettenmann, 2007). Based on this knowledge, an envisioned drug for TBI may be potent immunomodulatory proteins that could foster the therapeutic phagocytic activity of microglia.

## Redirecting Pro-Death Microglial Functions

Inactivating the pro-death inflammatory response of microglial cells is equally effective in combating the secondary cell death associated with TBI (Kapadia et al., 2008; Chen et al., 2011; Tsai et al., 2011). Recent pharmacologic strategies against TBI-induced secondary cell death employ inhibitors of oxidative stress and microglial activation. A high dose (100 mg/kg) pre-treatment with apocynin, an NADPH oxidase assembly inhibitor that retains proinflammatory profile of microglia, produces therapeutic potential against murine models of TBI (Choi et al., 2012; Zhang et al., 2012). One week after TBI, microglial activation remained, but ROS production was inhibited by apocynin in the hippocampal CA3 pyramidal neurons; they also found reduced BBB disruption, and neuronal rescue from cell death associated with TBI (Choi et al., 2012).

New therapies to attenuate exacerbation of microglia activation have emerged thanks to the study of potential biomarkers identified in models of acute spinal cord injury (SCI). Interestingly, an overlap in the cytokine profiles expressed by SCI rodents and human subjects has been recently demonstrated, in that rodent and human released in an SCI injury-dependent manner IL-6, IL-8, and monocyte chemoattractant protein 1 (MCP-1) (Stammers et al., 2012). A promising neuroprotective approach is the use of the antibiotic minocycline. In SCI animal models, minocycline has shown therapeutic effects in reducing microglial activation, excitotoxicity, and neuronal and oligodendrocyte cell death associated with mitochondrial stabilization (Teng et al., 2004; Festoff et al., 2006; Yune et al., 2007; Lee et al., 2012). Of note, a recent study demonstrated increased motor recovery in human patients suffering from acute SCI after 7 days of minocycline treatment relative to patients treated with the placebo drug (Casha et al., 2012) In the field of stem cell therapy, studies have also shown that a chemokine/cytokine (i.e., inflammatory) response may actually guide the migration of stem cells from the periphery to the site of brain injury, thereby allowing efficient brain bioavailability of the grafted cells’ secreted therapeutic molecules (Borlongan, 2011; Borlongan et al., 2011). Such inflammation-mediated cell migration suggest that a modest cytokine/chemokine upregulation aids stem cells in reaching their brain injured target areas. Recognizing the balance between proinflammatory and anti-inflammatory microglial function may provide new targets for arresting TBI-induced secondary cell death.

## Contemplating Microglia-Based Biomarkers for TBI

In parallel to developing treatments for TBI, utmost consideration for research investigations should be devoted to exploring biomarkers for TBI which will help optimize therapeutic intervention, in that the proper timing for treatment initiation will be guided by onset or peak time window of secondary cell death as may be captured by novel biomarker tools.

### Cytokine profiling

Cytokine profiling of microglial cells may lead to identification of specific proteins that regulate microglia (e.g., BDNF) and measuring these proteins in the blood or CSF may provide clues on the status of the TBI patient. Unraveling the cytokine and chemokines profile of activated microglia could lead to the identification of specific proteins that regulate microglia response after TBI (e.g., BDNF, NGF, and TGF-β). This approach would have to tackle the paracrine and autocrine roles, as well as interactions between cytokines, chemokines, and neurotrophic/tropic factors. Defining a cytokine profile for activated microglia in TBI can give us new insights on known neuroprotective approaches post-TBI. This knowledge may translate into the human clinical scenario by measuring these recognized proteins in the blood, plasma, or CSF and provide clues on the status of the TBI patient. For instance, cerebral microdialysis is a well-established laboratory tool that is increasingly used as a bedside monitor to provide on-line analysis of brain tissue biochemistry during neurointensive care (Tisdall and Smith, 2006). In a microdialysis study 12 patients suffering from diffuse severe TBI, defined as a post-resuscitation Glasgow Coma Score ≤8, were monitored over a period of 5 days. Their cerebral fluid, arterial and jugular venous plasma samples were screened for a cytokine and chemokine dataset using a principal component analysis and partial least squares discriminant analysis to demonstrate the pattern of production following TBI, distinct phases of the humoral inflammatory response and the differing patterns of response in brain and in peripheral blood (Helmy et al., 2012). Brain tissue microdialysis can become an established technique for monitoring acute and chronic TBI if future studies are capable of identifying an overlap in microglial cytokine profile against the microdialysis data analysis. Potential biomarkers identified in rat models of TBI that should be included in the repertoire of microglial profiling and overall brain tissue profiling by microdialysis may include epithelial/endothelial tyrosine kinase (Wu et al., 2012), poly(ADP-ribose) polymerase-1 (d’Avila et al., 2012), and myeloid differentiation primary response protein 88 (Li et al., 2011). In addition to this emerging cytokine profile, novel cytokines and associated proteins may be detected via high throughput screening assays (e.g., microRNA analysis) using blood, CSF, or tissue samples from TBI patients and animal models from time of impact and over different periods of secondary cell death evolution.

### Detecting phagocytic profile

Detecting phagocytic profile of microglia may reveal close molecular and cellular association between NPCs and stem cells. In addition, measuring levels of NPCs or stem cells via imaging modalities (functional MRI) may reveal the phagocytic activity of microglia. The identification of potent immunomodulatory proteins in the phagocytic profile of microglia, NPCs, and stem cells in general could help us elucidate the overlap in modulatory and phagocytic functions among these cell types. It seems tangible to measure levels of NPCs or stem cells via imaging modalities (e.g., functional MRI) to provide real-time status of the phagocytic activity of microglia (Yu et al., 2010). Likewise, we could attempt to correlate already known phagocytic biomarkers (e.g., LC3) with those of inflammation and apoptosis to establish a causal relationship among these three critical cellular processes in the TBI brain. For example, in a lateral moderate fluid percussion injury model of TBI in adult rats, microarray analyses revealed apparent time-dependent expression changes in 23 apoptosis-related genes, including inflammatory cytokines such as IL-1-α, IL-1-β, and TNF which immediately increased at 3 h following the injury (Shojo et al., 2010). Thus, these time-dependent gene expression profiles elucidate the progression of the secondary cell death process of apoptosis, shown in this study as an ensuing event associated with inflammation (Shojo et al., 2010).

### Assessing the level of “proinflammatory response of microglia”

Assessing the level of “proinflammatory response of microglia” could be measured via blood/CSF assays (Ziebell et al., 2011; Jin et al., 2012). The intent here is to visualize a threshold of pro-inflammation that is therapeutic (i.e., serving as signaling cue for stem cell migration from periphery to the injured brain), and has not reached a level that could invoke the deleterious neuroinflammatory response responsible for exacerbation of TBI pathological symptoms. For example, measuring *S*-nitrosoglutathione (GSNO), a nitrosylation-based signaling molecule, could reveal brain levels of peroxynitrite and oxidative metabolites, which when reduced levels are detected may indicate protection of the neurovascular unit integrity (Khan et al., 2009, 2011). Coincidentally, the detection of increased neurotrophic factors produced by constant low levels of GSNO treatment over time may represent enhanced synaptic plasticity (Khan et al., 2009, 2011). Accordingly, assessment of surrogate markers of GSNO involving peroxynitrite and oxidative metabolites, and neurotrophic factors may provide insights on neurovascular integrity and synaptic plasticity in TBI.

## Conclusion

The major pro-survival feature of microglia is their phagocytic activity. Identifying the signaling factors that nurture microglia to preserve their regenerative function after injury versus the predominating inflammatory activity (e.g., neuroinflammation) will provide insights into homeostatic mechanisms in maintaining a healthy brain. An appealing characteristic of microglia, which deserves more attention, is their migration to the site of injury. However, the migratory mechanisms thriving microglia to populate the CNS after arrival at the injured brain remain poorly understood. An in-depth examination of the molecular cues that regulate the anti-inflammatory response will guide the development of effective treatments to reduce detrimental effects of microglial activation and shift their function toward microglia-based therapies for TBI.

## Conflict of Interest Statement

The authors declare that the research was conducted in the absence of any commercial or financial relationships that could be construed as a potential conflict of interest.
